# Sex‐specific variation in thermal sensitivity has multiple negative effects on reproductive trait performance

**DOI:** 10.1111/1365-2656.70026

**Published:** 2025-03-18

**Authors:** Matilda Q. R. Pembury Smith, Daniela Trojmar, Karl Gotthard, Christer Wiklund, Rhonda R. Snook

**Affiliations:** ^1^ Department of Zoology Stockholm University Stockholm Sweden; ^2^ Bolin Centre for Climate Research Stockholm University Stockholm Sweden

**Keywords:** butterfly, climate change, fertility, reproduction, sperm heteromorphism, spermatogenesis

## Abstract

1. Understanding how increasing temperatures influence ectotherm population growth rate is necessary for predicting population persistence. Population growth rate depends on the thermal performance of multiple life‐history traits that have different thermal sensitivities. Reproductive traits are considered more thermally sensitive than other life‐history traits, such as survival and development rate. Moreover, the thermal sensitivity of reproductive traits can be sex‐specific, which may differentially affect population growth. However, research concurrently assessing the sex‐specific influence of heat stress on multiple reproductive traits is limited.
2. We investigated the effect of heat stress on pupal survival and reproductive traits in both sexes to determine sex‐specific thermal sensitivity and reproductive performance. Individuals of the butterfly *Pieris napi* were reared at either 22°C or 29°C throughout the larval and pupal stages. The latter temperature reflects the fastest development rate in this population, influencing generation time, a common population growth rate metric. We recorded pupal survival and adult body weight in both sexes. After eclosion, males and females from both treatments were allowed to interact, and mating success, copulation duration, egg production, fertility and male sterility recovery were measured. A subset of mated females was dissected to assess the number and length of fertilising eupyrene and non‐fertilising apyrene sperm transferred by males of each treatment.
3. While elevated temperatures reduced pupal survival and resulted in smaller body weights in both sexes, more substantial sex‐specific effects on reproductive traits were observed. Mating success was reduced in heat‐stressed females but not in males. In contrast, egg production and fertility were unaffected by heat stress in females, while heat‐stressed males, despite having longer copulation durations, exhibited near‐complete sterility. Male heat‐induced sterility was mediated by a disruption to both eupyrene and apyrene sperm production or transfer. Male remating did not recover fertility, suggesting continued negative effects on sperm production.
4. Our results highlight how increasing temperatures affect reproduction, illustrating that temperatures generating optimal performance for non‐reproductive traits, like development rate, can negatively and differentially impact sex‐specific reproductive fitness. These negative reproductive consequences may impact population persistence, highlighting the necessity to incorporate these findings into future advanced models predicting species' responses to climate warming.

## Graphical abstract

We show sublethal, sex‐specific effects on reproductive traits at a temperature that accelerates development, with female mating success severely reduced and male sterility nearly complete. Our results emphasise that temperatures maximising performance in one trait may negatively and differentially impact sex‐specific reproductive fitness, affecting population persistence and climate change predictions.

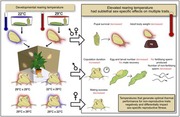

## 1 INTRODUCTION

Anthropogenic climate change is increasing both average local temperatures and the likelihood and duration of extreme weather events such as heatwaves (IPCC, 2014). Ectotherms, whose physiology and behaviour depend on ambient temperature, may be particularly vulnerable (Deutsch et al., 2008). The thermal tolerance of ectotherms can be described using thermal performance curves, which identify temperatures that result in peak trait performance (*T*_opt_) and the critical thermal limits (CTL; CT_min_/CT_max_)—the temperature boundaries beyond which critical biological functions fail or death occurs (lethal limits) (Angilletta, 2009).

The thermal performance of life‐history traits influences population growth rates. For example, warmer temperatures can increase development rates up to an optimum (i.e. fastest development), decreasing generation time, a common metric of intrinsic population growth rate (Kingsolver & Huey, 2008). However, different life‐history traits have trait‐specific thermal performance curves (Pawar et al., 2024). Notably, there is growing evidence that infertility occurs at temperatures below both CT_max_ (e.g. David et al., 2005; Parratt et al., 2021; Walsh et al., 2019), and *T*_opt_ for traits such as developmental rate (Pawar et al., 2024). Therefore, focusing on these traits, without accounting for the potential temperature gap between lethal and sublethal fertility effects, may underestimate the negative effects of a warming climate on ectotherms (Parratt et al., 2021; van Heerwaarden & Sgrò, 2021; Walsh et al., 2019).

Assessing responses to heat stress in both sexes is also crucial when making accurate population viability estimates, as several studies have shown sex‐specific differences in fertility following exposure to the same stressful thermal regime (e.g. Baur et al., 2022; Costa et al., 2023; Parrett et al., 2024; van Heerwaarden & Sgrò, 2021; Zwoinska et al., 2020). For instance, elevated temperatures disrupt male, but not female, reproductive output (offspring production) in the flour beetle *Tribolium castaneum* (Sales et al., 2018), and heat stress in females, but not males, reduces egg production in the butterfly *Bicyclus anynana* (Janowitz & Fischer, 2011). While these results indicate that the sexes may be differentially sensitive, such studies are quantitatively rare. A large systematic map of the literature on the effects of thermal stress on reproduction found that most research does not employ an experimental design capable of determining sex‐specific responses (Dougherty et al., 2024). Additionally, over 70% of these studies only assessed thermal effects on fecundity (the number of offspring produced by fertile individuals; Dougherty et al., 2024). Given that heat‐induced changes to mating behaviour and mating success can also impact population viability and performance, it is important to examine how temperature affects multiple aspects of reproduction in both sexes to better predict long‐term climate change impacts (Leith et al., 2022; Pilakouta & Baillet, 2022).

The effect of high temperatures may also vary depending on the timing of heat stress in relation to life‐history stage. For some organisms, such as holometabolous insects, development may be more sensitive to increased temperatures than the adult phase, as movement from unfavourable temperatures is restricted and often limited to a food resource (Kingsolver et al., 2011). Heat‐induced disruptions to development can affect adult phenotypes (Eyck et al., 2019), such as reducing adult body weight (e.g. Forsberg & Wiklund, 1988; Kingsolver & Huey, 2008), energy reserves (e.g. Greenleaf et al., 2007) and morphological features such as antennae length (Moradinour et al., 2023), which could impact reproductive success. Additionally, gametogenesis occurs during juvenile developmental stages in many species, with heat stress subsequently causing adult sterility (e.g. David et al., 2005; Porcelli et al., 2017; Rodrigues et al., 2022; Sales et al., 2021; Walsh et al., 2021; Zwoinska et al., 2020). Therefore, to make better‐informed climate response predictions, the effect of elevated temperatures during juvenile stages on multiple adult reproductive traits should be examined.

Lepidoptera are a model taxon for examining the effects of climate change (Warren et al., 2001), being among the first taxa for which evidence of both climate change‐induced range shifts (Parmesan, 1996; Settele et al., 2008) and phenology (Sparks & Yates, 1997) was documented. Here, we use the widespread and common butterfly *Pieris napi* L. (Lepidoptera: Pieridae). Species in the *Pieris* genus serve as both important pollinators (Goulson & Cory, 1993) and pest species (Ryan et al., 2019). Previous studies on *P. napi* show that elevated temperatures increase the development rate, but subsequently increase mortality and decrease body size (Moradinour et al., 2023; von Schmalensee et al., 2021, 2023). Since male body size positively correlates with ejaculate size (Larsdotter Mellström & Wiklund, 2015; Wiklund & Kaitala, 1995), higher temperatures may limit reproduction. However, the sex‐specific effects of developmental temperature on reproductive phenotypes remain unexplored in this species.

To address this knowledge gap, we conducted thermal manipulation experiments to test sex‐specific effects on reproductive traits and pupal survival (eclosion success), which allow us to examine potential differences in survival and fertility limits in this species. Survival responses to heat stress during the pupal stage are more sensitive than other developmental stages in Lepidoptera, likely due to restricted movement (Liu et al., 2002; Long et al., 2016). Thus, pupal survival acts as a robust proxy for comparing survival and fertility. Our experiment included a benign control treatment of 22°C that lies within the range of temperatures that this species undergoes direct development (Larsdotter Mellström & Wiklund, 2015; Prasai & Karlsson, 2012), and an elevated temperature treatment of 29°C that represents *T*_opt_ for both larval and pupal development rates in the studied population (Moradinour et al., 2023; Siemers et al., 2024; von Schmalensee et al., 2023). These treatments also span within the average temperature (18.5°C) and the average maximum temperature (30°C) recorded in June in Stockholm, Sweden (the period during which the experiment was conducted; TAD, 2024), when most *P. napi* individuals are undergoing development (von Schmalensee et al., 2023). Both sexes were subjected to each temperature treatment to determine the thermal sensitivity of pupal survival, body weight and a number of reproductive traits such as mating success, copulation duration (of an initial mating), egg production, fertility and the potential for males to recover fertility following an additional mating. As Lepidoptera are sperm heteromorphic, producing both short, non‐fertilising apyrene sperm that lack DNA and long, fertilising eupyrene sperm (Friedländer, 1997; Wedell & Cook, 1999), we also investigated a potential mechanism underlying heat‐induced male sterility by examining heteromorphic sperm production and morphology. Overall, our research provides new insights on the sex‐specific sensitivity of reproductive traits at a temperature that maximises developmental rates, suggesting the potential for negative, rather than positive, impacts on population dynamics as temperatures rise.

## 2 METHODS

### 2.1 Ethics declaration

No ethical approval was required for this work.

### 2.2 Study organism

*Pieris napi* L. (Lepidoptera: Pieridae) is a widespread and common butterfly species found in Europe, Asia and North America (GBIF, 2024). This is a protandrous species, where males eclose before females. Males transfer a spermatophore to females during mating. The spermatophore makes up 15% of male body weight (Kaitala & Wiklund, 1994) with the nutritional content partly incorporated by females into eggs (Bergström & Wiklund, 2002) and the sperm component containing heteromorphic sperm (Cook & Wedell, 1999; Swallow & Wilkinson, 2002). Spermatogenesis in *P. napi* has not been investigated, however, studies on other Lepidopteran species have shown that spermatogenesis of each sperm type is sequential, with eupyrene sperm production beginning during the larval phase and apyrene spermatogenesis beginning close to pupation (Friedländer, 1997; Friedländer et al., 2005). Female *P. napi* are typically polyandrous (Andersson et al., 2004) and males require 2 days after their first mating to produce another spermatophore of the same size and quality as the initial mating due to ejaculate reserve depletion (Bissoondath & Wiklund, 1996). After mating, females oviposit individual eggs on host plants that provide a food source for developing larvae. This species is oligophagous, using members of the Brassicaceae family, and displays equivalent larval performance on a range of hosts (Friberg et al., 2015; Friberg & Wiklund, 2019).

### 2.3 Insect rearing

**FIGURE 1:**
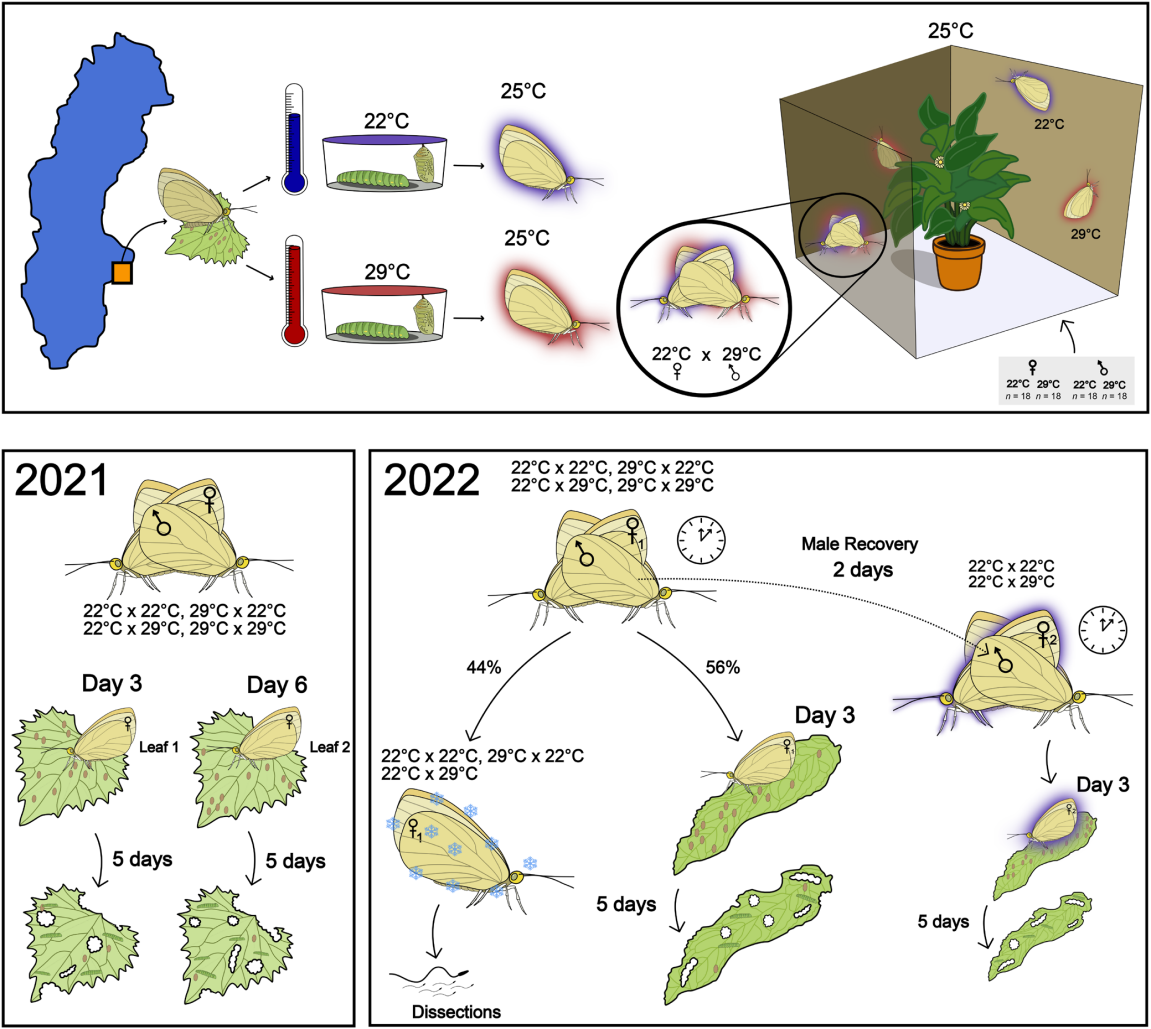
A schematic of the experimental design conducted in 2021 and 2022. In both years, wild‐caught individuals were collected from Stockholm, Sweden. Females oviposited on a host plant. Once hatched, larvae were moved into one of two temperature treatments, a benign 22°C (blue) or warm 29°C treatment (red) until eclosion. After eclosion, 18 adult males and females from each thermal treatment were introduced into a mating area to mate freely, generating four possible mating combinations: 22°C × 22°C, 22°C × 29°C, 29°C × 22°C, 29°C × 29°C (female treatment always listed first). In 2021, mated females oviposited for 3 days after which they were transferred to a new leaf to oviposit for another 3 days and subsequently discarded. Eggs produced between day 1–3 were counted on day 3 and eggs produced between day 4–6 were counted on day 6. Larvae were counted 5 days after egg counts. In 2022, copulation duration (indicated by the clock symbol) was recorded for all matings. Following copulation, a subset of the females (44%) were immediately frozen and dissected to conduct sperm counts, while the remaining mated females were left to oviposit for 3 days and subsequently discarded. Egg and larval counts were conducted following the same protocol as 2021. Males that mated with females were also allowed to remate with a virgin 22°C female, 2 days after the male's first mating to examine male recovery. Copulation duration was recorded, and mated females were processed for egg and larval counts following the same protocol as the initial experiment.

We report on two experiments: one from July–October 2021 and another from August–December 2022. Wild‐caught females, known to be mated (Svärd & Wiklund, 1989; Wiklund & Forsberg, 1991), were randomly captured in flight in Stockholm, Sweden (WGS84 decimal: Lat. 59.368, Long. 18.061) from July–August in 2021 and in August in 2022. Individuals were immediately put into flight cages (80 × 80 × 55 cm) at 25°C (8L:16D) and lit from above using daylight mimicking lamps for 8 h/day between 09:00 and 17:00. This temperature was used to facilitate oviposition on the host plant (Figure 1; details below) provided in the flight cages. In addition to the host plant, each cage contained a potted *Kalanchoe* sp., augmented with a 25% sucrose solution applied once daily for feeding. In order to maintain high humidity, the bottoms of the cages were covered in paper towels that were wetted at least once a day, and cages were sprayed with water every few hours during daylight hours. At the end of each day, host plant leaves with oviposited eggs were placed in bottles within cages and moved into a larger breeding room under 22L:2D day lengths at 22°C, an environmental treatment that generates direct development (Larsdotter Mellström & Wiklund, 2015). In 2021, hatched eggs from wild‐caught females were transferred to experimental treatments (see next section). In 2022, to standardise maternal effects, we used F2s. F1s continued developing in the breeding room at 22°C and were provided ad libitum with host plant leaves during development. Subsequent F1 adult males and females were placed in flight cages, where mating and oviposition occurred. The F2 oviposited eggs were then treated as described for 2021.

### 2.4 Experimental treatments

See Figure 1 for a summary of experiments performed. Experimental first instar larvae reared as eggs at 22°C were put into either 22°C or 29°C treatments throughout development. In 2021, we reared experimental larvae in plastic containers (5/container) with a hole in the bottom set into another, narrower, 1 L container (∅ 12 cm, 12.5 cm high) filled with water to keep the leaves of the host plant, *Allaria petiolata*, hydrated to maintain humidity. Each container was covered with a net and fastened with a rubber band to prevent individuals from escaping. In 2022, we mass reared experimental larvae in cages (34 × 40 × 46 cm) with the host plant *Armoracia rusticana* in bottles with water to maintain humidity. Host plant species varied between years given differences in the timing of the experiment and therefore the abundance of available host plants. Additional host plant leaves were provided as needed until pupation. In both years, pupae were transferred to individual containers (∅ 4.4 cm, 4 cm high) and kept at their development temperature treatment until eclosion. As larvae reared at 29°C develop faster than those reared at 22°C, larvae to be reared at 29°C were transferred to this temperature treatment 6 days after larvae were transferred to the 22°C treatment to ensure individuals kept at both treatments eclosed at approximately the same time. Individuals that either did not eclose or failed to eclose properly (e.g. not able to fly) were sexed and used to calculate temperature effects on pupal survival. Successfully eclosing individuals were weighed to the nearest 0.1 mg (Precisa 205A SCS balance), uniquely numbered with a marker pen to allow individual identification, and used in subsequent experiments. These adults were initially kept at 8°C–10°C to slow ageing (Angilletta, 2009), until a sufficient sample size had been collected for experimentation (1–3 days for males, 1–5 days for females). Individuals that had eclosed the closest to the start of the experiment, and equal numbers of individuals from each day of eclosion, were chosen for the experiment.

For both 2021 and 2022, after eclosion, 18 adult males and females from each thermal treatment (72 per cage; Figure 1) were transferred into separate flight cages (*n* = 4 in 2021, *n* = 3 in 2022; 80 × 80 × 55 cm) maintained at 25°C. Each cage contained potted *Kalanchoe* sp., which was watered to maintain humidity and supplemented with drops of 25% sugar solution. Males were transferred to the cages 2 days before females, given that this species is protandrous (Bissoondath & Wiklund, 1996). After 2 days, females were put into the cages to mate freely. Thus, we conducted a choice test, a classic mate choice experimental design (Dougherty & Shuker, 2015) that reflects the sexual interactions that this species experiences in nature. In doing so, we produced four possible mating pairs within each cage: 22°C × 22°C, 22°C × 29°C, 29°C × 22°C, 29°C × 29°C (Figure 1).

For both years, besides measuring eclosion success and body weight as described above, we also measured mating success, and following a mating, the number of eggs produced and the larval number that subsequently hatched (fertility). In 2021, egg production and fertility were measured at two time points: day 3 (which represents the eggs laid from days 1–3 following copulation after which the female was moved to a new host plant), and day 6 (which represents the eggs laid from days 4–6 following copulation) (Figure 1). In 2022, both egg and larval number were measured again but only at one time point (Figure 1; days 1–3 = day 3), as we found fertility consequences did not vary across days in 2021. In 2022, a proportion of mated females, either with 22°C or 29°C mates, were sacrificed immediately after mating to assess both sperm numbers and length transferred by males from the different thermal treatments (Figure 1). Additionally, following an initial copulation, we also measured whether males recover from heat‐induced sterility. Mated males were placed into flight cages without females for 2 days to potentially recover ejaculates, and then virgin control 22°C females were added to the flight cages. Copulation duration, egg production and fertility were measured and subsequently tested for the effect of temperature on recovery (Figure 1). See below for individual sections describing how we measured and analysed these traits.

### 2.5 Trait measurements and statistical analysis

All analyses were performed using R v 4.2.2 (R Core Team, 2020). The ‘lme4’ v 1.1‐34 and the ‘VGAM’ v 1.1‐11 packages were used to generate statistical models, and the ‘ggplot2’ package v 3.4.3 was used to generate all figures (Wickham, 2016). For all traits in which the relationship between temperature and sex was examined, the interaction between these variables was included in the model. Main effects, interactions and covariates were evaluated using the *Anova()* function (type III) from the ‘car’ package v 3.1‐2. If significant, post hoc comparisons of main level effects and interactions were performed using the contrast function in *emmeans* from the ‘emmeans’ package v 1.8.8. When additional variables were included in the model, model comparisons were conducted using log‐likelihood ratio tests to select minimal adequate models. Estimates and significance values for main effects and covariates are from minimal adequate models which are described below. When mating episode was examined in the model, no model reduction was conducted as understanding the interaction between mating episode and the other fixed effects was a key aim of the analysis. For full details on all models (Table S1), model selection (Tables S2–S6), and summary statistics (Table S8) see Supporting Information.

#### 2.5.1 Pupal survival and body weight

To examine the effect of the 29°C treatment on pupal survival, the proportions of individuals that eclosed as adults following pupation were analysed using a generalised linear model with a logit‐linked binomial distribution. The response variable was entered as a paired variable ‘cbind(eclosed, not eclosed)’ (Thomas & Lello, 2017). Sex (male or female), temperature (22°C or 29°C), their interaction and experimental year (2021 or 2022) were included as fixed effects (Table S2).

To analyse the effect of developmental temperature on adult body weight and to test whether this varied between the sexes, we used a linear model. Adult body weight was included as the continuous response variable, and temperature (22°C or 29°C), sex (male or female), their interaction, and experimental year (2021 and 2022) were included as categorical fixed effects (Table S3).

#### 2.5.2 Mating success and copulation duration

In both years, flight cages were monitored for mating every 10 min. Copulating pairs were caught and put into plastic containers (∅ 9 cm, 11 cm high) with a net covering the top until copulation ended. No individuals decoupled during or immediately after this procedure. Individual IDs of each copulating pair were noted. The first 50% of pairs that mated across all cages (18 of each sex from each thermal treatment/cage) were used to test whether the rearing temperature of either sex significantly affected the frequency of mating. This design stratifies the data to include chosen pairs rather than those that mated with the remaining available mates (Gilbert & Starmer, 1985). We continued to capture and record further matings in the cages, and all mated females were used for subsequent egg and larval counts (see Section 2.5.3).

To determine the effects of temperature on mating success, we used a generalised Poisson regression model to account for under‐dispersion. Within each flight cage, the frequency of pairs that mated from each mating combination (22°C × 22°C, 22°C × 29°C, 29°C × 22°C, 29°C × 29°C) was noted up until 50% of individuals had mated. Mating frequency was then included as the response variable, and male temperature (22°C and 29°C), female temperature (22°C and 29°C), their interaction and experimental year (2021 and 2022) were included as fixed effects (Table S4).

In 2022, copulation duration was examined using a linear mixed model. Male temperature (22°C and 29°C), female temperature (22°C and 29°C) and their interaction were included as fixed effects. Male weight and female weight were included as covariates. Flight cage was included as a random effect.

#### 2.5.3 Egg number and fertility

After mating, individual females were put on a host plant leaf, and the number of eggs oviposited and the larval number that subsequently hatched were counted. Counts occurred on day 3 (eggs laid from days 1–3 following copulation) and day 6 (eggs laid from days 4–6 following copulation). We used linear models to examine the effect of temperature stress in each sex on the response variables of egg production and fertility in two different analyses: (i) determining whether measurement day (3 vs. 6) varies (2021 data only; Table S5) and (ii) assessing whether day 3 data vary between experimental years (2021 vs. 2022; Table S6). For all models, male temperature (22°C and 29°C), female temperature (22°C and 29°C) and their interaction were included as fixed effects, and male and female weight as covariates. For the fertility analysis, we also included the number of eggs produced as a covariate. For (i) we also included measurement day (3 vs. 6) and for (ii) experimental year (2021 and 2022) as fixed effects. Other than larval number in model (ii), all response variables were square root transformed to ensure normality.

#### 2.5.4 Male recovery and copulation duration

Given we found that males reared at 29°C are typically sterile and copulating with them reduces female egg production, in 2022 we examined whether males could recover fertility following a second mating. Following the mating success experiment (see Section 2.5.2), mated males (reared at either 22°C or 29°C) were placed into a male‐only flight cage immediately after the initial copulation ended, with 22°C and 29°C males in separate cages. Cages were provided with potted *Kalanchoe* sp. which were watered to maintain humidity and supplemented with drops of 25% sugar solution. Equal numbers of control virgin 22°C females were then added to each cage, 2 days after the males' initial mating. Cages were monitored every 10 min, and once a copulation occurred, pairs were placed into small containers and copulation duration was recorded. Once remated, males were discarded and mated females were kept for egg and larval counts as described in Section 2.5.3.

To compare the effect of temperature on copulation duration, egg production and fertility between an initial mating and a male's second mating, linear mixed models were performed. As the primary aim was to assess temperature effects across mating episodes, all analyses included a three‐way interaction between mating episode (initial mating and remating), male temperature (22°C and 29°C) and the temperature of the male's first mate (22°C and 29°C). The latter variable is included to account for any strategic allocation of ejaculates that may affect subsequent fertility (Larsdotter Mellström & Wiklund, 2009; Larsdotter Mellström & Wiklund, 2015). For all models, male weight and female weight (male's second mate) were included as covariates, and male identity was included as a random effect to account for repeated measures. Egg and larval numbers were square root transformed to ensure normality. When examining fertility, egg number was also included as a covariate. Sample sizes for the effect of mating episode on copulation duration are larger than for egg production and larval number, given half of the females from the first mating episode were sacrificed for sperm analysis (Table S8).

#### 2.5.5 Eupyrene and apyrene sperm number and length

To examine how developmental heat stress impacts sperm, a subset of mated females in 2022 was sacrificed by decapitation within 5 min of the termination of copula, before sperm migration from the spermatophore, and frozen at −20°C. These females were subsequently dissected to count the number of eupyrene and apyrene sperm and measure sperm length. Individual females were thawed, and the intact spermatophore was removed from the female bursa and isolated in phosphate‐buffered saline. The ampulla where the sperm is contained was ruptured, and the sperm mass was dispersed by gentle stirring. The eupyrene bundles, each consisting of 256 eupyrene sperm (Gage & Cook, 1994), were counted under 40× magnification. The dispersed solution was washed into a 30‐mL specimen tube with phosphate‐buffered saline and diluted with distilled water to 15 mL. Six 10‐μL subsamples of each diluted sample were air‐dried on glass slides, and the number of apyrene sperm in each dried 10‐μL aliquot was counted under ×100 magnification using dark‐field phase contrast microscopy (Wedell & Cook, 1999). The numbers of apyrene sperm per ejaculate were calculated by multiplying the average sperm number of the six 10‐μL subsamples by its dilution factor. Due to a lack of eupyrene sperm produced by males reared in the 29°C treatment, length measurements were only carried out on apyrene sperm. One to two individual apyrene sperm from each of the six 10‐μL subsamples were photographed (Cannon 600D) at ×200 magnification, producing 6–8 photographs from each individual dissected female. For some individuals, fewer than six apyrene sperm were present across all six 10‐μL subsamples. In these cases, photographs were taken of all apyrene sperm present to measure sperm length. The length of each apyrene sperm was measured using ImageJ (Abramoff et al., 2004).

To examine the effect of temperature on eupyrene and apyrene sperm number, linear models were performed, and to examine the effect of temperature on apyrene sperm length, a linear mixed model was used to account for male identity as a random effect due to repeated measures. In all models, male temperature (22°C and 29°C) and female temperature (22°C and 29°C) were included as fixed effects. No interaction term was examined as no pair between a male reared at 29°C and a female reared at 29°C was dissected. Male and female body weights were included as covariates. All response variables were square root transformed to ensure normality.

## 3 RESULTS

### 3.1 Elevated temperature reduces pupal survival

**FIGURE 2:**
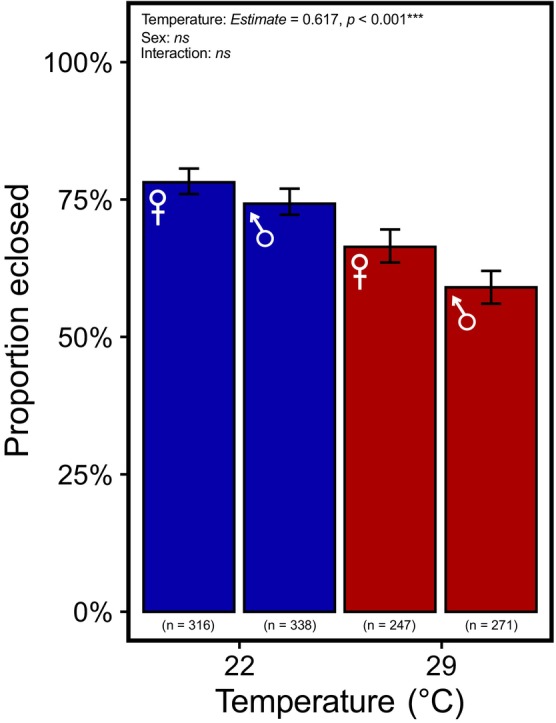
The effect of developmental rearing temperature on pupal survival. Individuals were exposed to two different developmental temperatures (22°C and 29°C). The proportion of individuals that successfully eclosed to adults following pupation was measured and their sex noted. The numbers at the bottom of each bar represent the total sample size for each sex at each temperature treatment across both measurement years. Colour represents developmental rearing temperature.

Exposure to the 29°C treatment reduced viability, reflecting harmful temperature stress. However, a large fraction of individuals survived to adulthood in both treatments (Figure 2; 63% at 29°C and 76% at 22°C). Individuals reared in benign conditions had significantly higher pupal survival compared to individuals reared in heat‐stressed conditions (Figure 2; odds ratio: 1.9; *Estimate* = 0.617 ± 0.131, *z* = 4.718, *p* < 0.001). While pupal survival was marginally higher in 2022 compared to 2021 (*Estimate* = 0.255 ± 0.132, *z* = 1.935, *p* = 0.053), sex (ANOVA: *χ* ^2^ = 1.306, df = 1, *p* = 0.253), the interaction between sex and year (ANOVA: *χ* ^2^ = 0.209, df = 1, *p* = 0.647), and a three‐way interaction between temperature, sex and year (likelihood‐ratio test: *p* = 0.603) did not significantly affect pupal survival.

### 3.2 Elevated temperature reduces adult body weight in both sexes

Elevated developmental temperature significantly reduced adult body weight (*Estimate* = −0.005 ± 0.001, *t* = −4.563, *p* < 0.001), with individuals reared at 29°C showing a 6% reduction compared to individuals reared at 22°C. The effect of temperature on adult body weight was the same for both sexes since there was no significant interaction between temperature and sex (ANOVA: *F* = 1.387, df = 1, *p* = 0.240), and there was no significant three‐way interaction between temperature, sex and year (likelihood‐ratio test: *p* = 0.371). This pattern suggests that this relationship was consistent across years, even though body weight was significantly higher in 2021 than in 2022 (*Estimate* = 0.003 ± 0.001, *t* = 3.371, *p* < 0.001). Males were significantly heavier than females (*Estimate* = 0.004 ± 0.001, *t* = 3.926, *p* < 0.001).

### 3.3 Elevated temperature reduces female mating success

**FIGURE 3:**
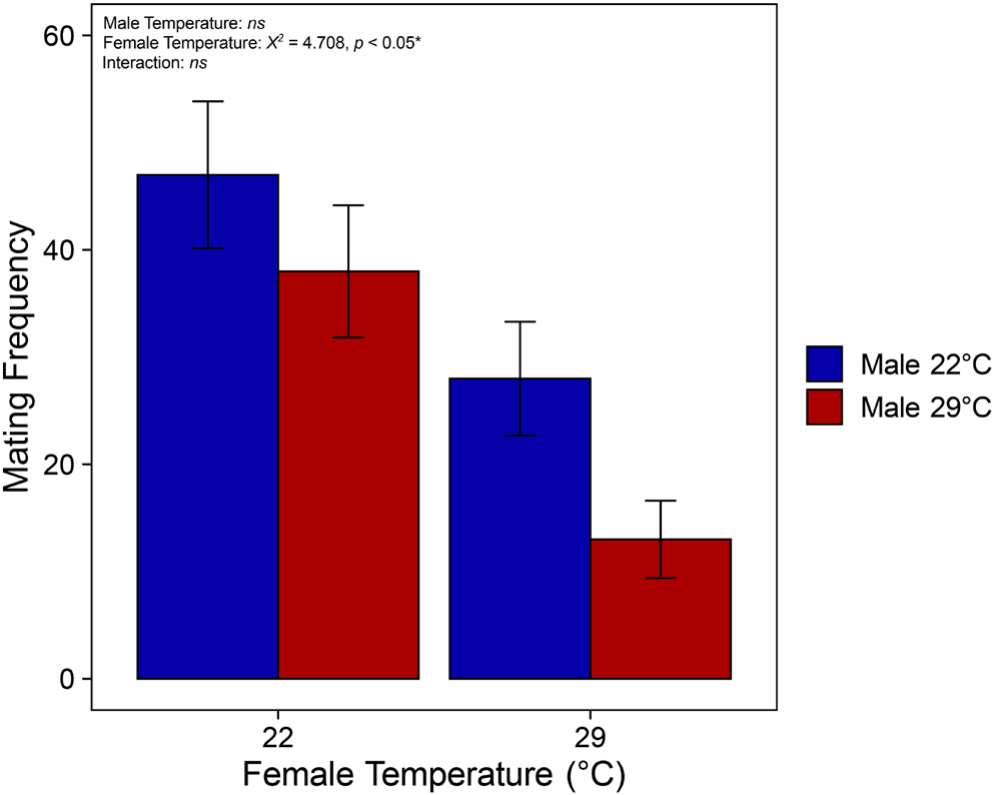
The effect of developmental rearing temperature on mating success. The frequency of mating males and females from each temperature treatment in the first 50% of copulations. Only the first 50% of copulations were analysed, as this stratifies the data to include pairs that could choose partners rather than those that mated with the remaining available mates. Male rearing temperature is denoted by colour.

Females reared at 29°C engaged in significantly fewer copulations than females reared at 22°C (Figure 3; ANOVA: *χ* ^2^ = 4.708, df = 1, *p* < 0.05). There was no significant effect of male temperature (ANOVA: *χ* ^2^ = 0.950, df = 1, *p* = 0.330) or the interaction between male and female temperature (ANOVA: *χ* ^2^ = 1.921, df = 1, *p* = 0.166). There was no significant three‐way interaction between male temperature, female temperature and year (likelihood‐ratio test: *p* = 0.800), suggesting that the relationship between male and female temperature on mating success was consistent across years.

### 3.4 Copulation duration increases when males, but not females, are reared at an elevated temperature

Male rearing temperature had a marginally non‐significant effect on copulation duration (ANOVA: *χ* ^2^ = 3.354, df = 1, *p* = 0.067), with males reared at 29°C displaying an 18% increase in copulation duration relative to males reared at 22°C. Neither female temperature (ANOVA: *χ* ^2^ = 0.016, df = 1, *p* = 0.901) nor the interaction between male and female temperature (ANOVA: *χ* ^2^ = 0.012, df = 1, *p* = 0.914) were significant.

### 3.5 Consistent negative effects on egg production and fertility are mediated by male exposure to elevated temperature

In the 2021 experiment, we found that females mated with males reared at 22°C oviposited significantly more eggs than females mated with males reared at 29°C (*Estimate* = 1.770 ± 0.530, *t* = 3.345, *p* < 0.01). Day significantly impacted egg number (more eggs were laid on days 3–6 compared to days 1–3; *Estimate* = 1.310 ± 0.480, *t* = 2.740, *p* < 0.01). Egg number was not affected by either female temperature (ANOVA: *F* < 0.001, df = 1, *p* = 0.996), the interaction between male and female temperature (ANOVA: *F* = 1.697, df = 1, *p* = 0.195) or a three‐way interaction between male temperature, female temperature and day (likelihood‐ratio test: *p* = 0.287).

After accounting for the number of eggs produced, in 2021, males reared at 29°C produced significantly fewer larvae than males reared at 22°C (*Estimate* = −3.660 ± 0.230, *t* = −15.891, *p* < 0.001). There was also a significant effect of measurement day (more larvae hatched on days 3–6 compared to days 1–3; *Estimate* = 0.598 ± 0.200, *t* = 3.000, *p* < 0.01). However, there was no significant effect of female temperature (ANOVA: *F* = 0.335, df = 1, *p* = 0.563), the interaction between male and female temperature (ANOVA: *F* < 0.001, df = 1, *p* = 0.999) or a three‐way interaction between male temperature, female temperature and day (likelihood‐ratio test: *p* = 0.426). Thus, temperature effects on fertility result from negative impacts on males.

**FIGURE 4:**
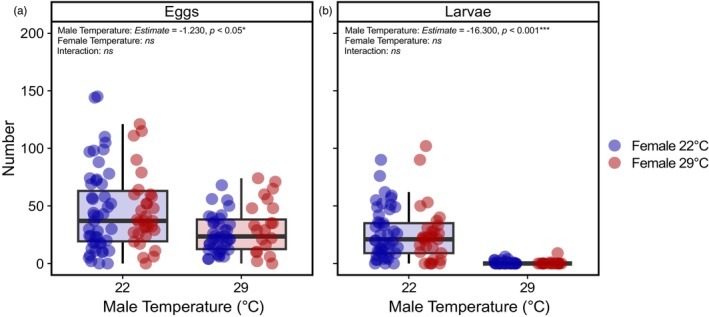
The effect of male developmental rearing temperature on (a) egg production and (b) larval production (fertility) across 3 days. In both 2021 and 2022, males and females were reared at two different developmental temperatures (22°C and 29°C) and allowed to mate freely. The number of eggs and larvae (fertility) produced 3 days post‐copulation was measured. Boxplot colour represents male rearing temperature; point colour represents female rearing temperature.

We next examined the consistency of the male effect on egg production and fertility by comparing equivalent data for the first 3 days post‐copulation from the experiments in 2021 and 2022. For egg production and fertility, there was no three‐way interaction between male temperature, female temperature and year (egg production: likelihood‐ratio test: *p* = 0.518; fertility: likelihood‐ratio test: *p* = 0.086), no significant interaction between male and female temperature (egg production: ANOVA: *F* = 0.289, df = 1, *p* = 0.592; fertility: ANOVA: *F* = 0.001, df = 1, *p* = 0.974), and no significant main effect of female rearing temperature on either trait (egg production: ANOVA: *F* = 0.271, df = 1, *p* = 0.604; fertility: ANOVA: *F* = 0.637, df = 1, *p* = 0.426). Year did not affect egg production (ANOVA: *F* = 0.203, df = 1, *p* = 0.653) but did influence fertility, with females mated to males reared at 22°C having slightly higher fertility in 2022 compared to 2021 (ANOVA: *F* = 4.026, df = 1, *p* = 0.047). The main impact for both egg production and fertility was male temperature treatment. Across both years, females mated with 29°C‐reared males produced 39.2% fewer eggs (Figure 4a: *Estimate* = −1.230 ± 0.442, *t* = −2.77, *p* < 0.05) and had a 97.7% reduction in larval production (Figure 4b: *Estimate* = −16.300 ± 2.320, *t* = −7.036, *p* < 0.001) relative to females mated with males from the 22°C treatment. Thus, male heat stress consistently negatively impacted fertility.

### 3.6 Copulation duration increases following remating

For males that remated, copulation duration displayed a 40% increase during the second mating compared to the initial mating (*Estimate* = 45.400 ± 9.860, *t* = 4.598, *p* < 0.001). However, temperature did not differentially affect this response since there was no significant interaction between mating episode and male temperature (ANOVA: *χ* ^2^ = 0.251, df = 1, *p* = 0.617), the temperature of the male's first mate (ANOVA: *χ* ^2^ = 1.108, df = 1, *p* = 0.292), or the three‐way interaction between male temperature, the temperature of the male's first mate and mating episode (ANOVA: *χ* ^2^ = 1.337, df = 1, *p* = 0.248). For the full model output, see Table S7.

### 3.7 Males do not recover fertility across two matings

There was a significant interaction between mating episode and male rearing temperature on female egg production (ANOVA: *χ* ^2^ = 4.343, df = 1, *p* < 0.05). Females mated to male's reared at 29°C produced significantly fewer eggs than females mated to male's reared at 22°C during the second mating (*Estimate* = −3.760 ± 1.140, *t* = −3.291, *p* < 0.01), whereas this effect was marginally non‐significant during the initial mating (*Estimate* = −2.100 ± 1.140, *t* = −1.841, *p* = 0.08). There was no significant interaction between the temperature of the male's first mate and mating episode (ANOVA: *χ* ^2^ = 1.228, df = 1, *p* = 0.268). The three‐way interaction between male temperature, the temperature of the male's first mate and mating episode was marginally non‐significant (ANOVA: *χ* ^2^ = 3.257, df = 1, *p* = 0.071). Neither the rearing temperature of the male's first mate (ANOVA: *χ* ^2^ = 0.053, df = 1, *p* = 0.818) nor their interaction with male rearing temperature (ANOVA: *χ* ^2^ = 0.174, df = 1, *p* = 0.676) had a significant effect.

**FIGURE 5:**
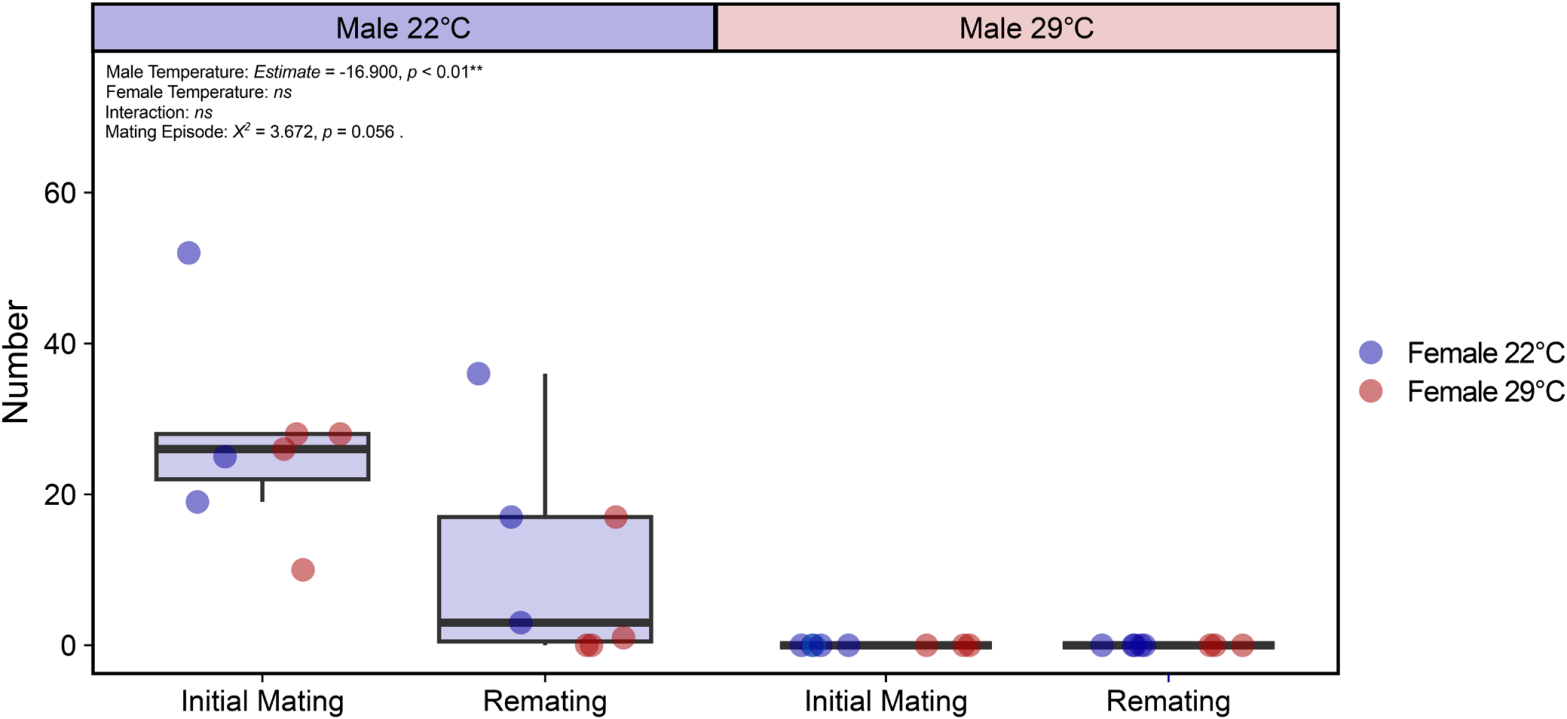
The effect of developmental rearing temperature on larval number produced following an initial mating and a second mating. Males (22°C or 29°C) mated to females (22°C or 29°C) in 2022 were given the opportunity to remate once with a virgin 22°C female 2 days after the males' first mating. Larval number (fertility) was measured to determine whether remating influences male fertility recovery. Boxplot colour represents male rearing temperature; point colour represents initial female rearing temperature.

Male mating episodes had a marginally non‐significant effect on fertility (Figure 5; ANOVA: *χ* ^2^ = 3.672, df = 1, *p* = 0.056). This pattern was driven by more offspring being produced during the initial mating compared to the second mating when the male was initially reared at 22°C, with no recovery observed when a male was initially reared at 29°C. There was no significant interaction between mating episode and male rearing temperature (ANOVA: *χ* ^2^ = 2.431, df = 1, *p* = 0.119) or the rearing temperature of the male's first mate (ANOVA: *χ* ^2^ = 0.137, df = 1, *p* = 0.711), and no significant three‐way interaction between male temperature, the rearing temperature of the male's first mate and mating episode (ANOVA: *χ* ^2^ = 0.017, df = 1, *p* = 0.897). As before, females mated to males reared at 29°C produced significantly fewer larvae compared to those reared at 29°C (Figure 5; *Estimate* = −16.900 ± 4.820, *t* = −3.512, *p* < 0.01), and there was no significant effect of the rearing temperature of the male's first mate (ANOVA: *χ* ^2^ = 1.227, df = 1, *p* = 0.268) or their interaction with male rearing temperature (ANOVA: *χ* ^2^ = 0.876, df = 1, *p* = 0.349).

### 3.8 Elevated temperature disrupts eupyrene sperm production or transfer and reduces apyrene sperm numbers and length

**FIGURE 6:**
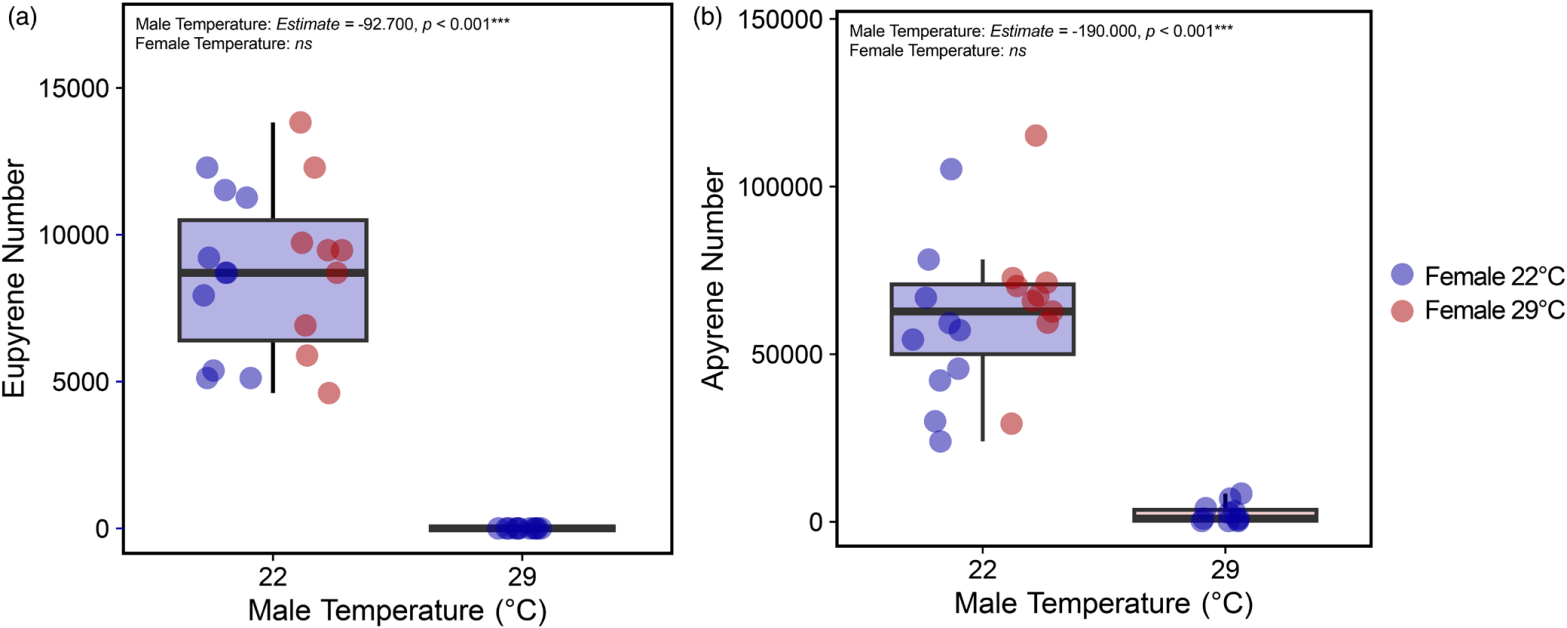
The effect of developmental rearing temperature on the number of (a) eupyrene and (b) apyrene sperm. A proportion of the females (22°C or 29°C) that mated with males (22°C or 29°C) in 2022 was immediately frozen following copulation and dissected. No pairs between males reared at 29°C and females reared at 29°C were dissected. Eupyrene and apyrene sperm numbers were measured. Each data point represents an individual cross. Box plot colour represents male rearing temperature, and point colour represents female rearing temperature.

Males reared at 29°C produced significantly fewer eupyrene sperm than males reared at 22°C (Figure 6a; *Estimate* = −92.700 ± 5.500, *t* = −16.852, *p* < 0.001). In fact, no eupyrene sperm were produced by males reared at 29°C, meaning that length measurements could not be compared between treatment groups. Female temperature treatment did not have a significant effect on eupyrene sperm number (ANOVA: *F* = 0.101, df = 1, *p* = 0.753). Males reared at 29°C produced significantly fewer (Figure 6b; *Estimate* = −190.000 ± 18.600, *t* = −10.180, *p* < 0.001) and shorter (*Estimate* = −0.245 ± 0.021, *t* = −11.630, *p* < 0.001) apyrene sperm than males reared at 22°C. Female temperature treatment did not have a significant effect on apyrene sperm number (*Estimate* = 27.500 ± 19.400, *t* = 1.419, *p* = 0.168) or length (*Estimate* = 0.007 ± 0.020, *t* = 0.319, *p* = 0.752).

## 4 DISCUSSION

Reproductive traits may exhibit greater thermal sensitivity than other life‐history traits, such as development rate and survival. Such differential sensitivity may significantly affect predictions of population resilience to rising temperatures (Parratt et al., 2021; van Heerwaarden & Sgrò, 2021; Walsh et al., 2019). Additionally, temperatures that appear to maximise somatic traits (e.g. development rate) can have negative fitness consequences at other life‐history stages or on different traits. Sex‐specific variation in how temperature affects reproductive traits may also differentially impact reproductive output (Iossa, 2019). However, the majority of research assessing the effects of temperature on reproduction has not used temperatures that optimise other life‐history traits, experimental designs capable of discerning sex‐specific thermal sensitivity, and has focused on a single reproductive trait—fecundity (Dougherty et al., 2024).

In this study, we delineate sex‐specific effects on pupal survival and reproductive traits by comparing juvenile exposure to a benign temperature treatment with a high‐temperature treatment that promotes the fastest development rate in *P. napi* (Moradinour et al., 2023; von Schmalensee et al., 2023). Heat stress decreased pupal survival—a stage particularly sensitive to heat‐induced mortality in Lepidoptera (Liu et al., 2002; Long et al., 2016)—and reduced adult body weight, which could further impact post‐eclosion survival (e.g. Doležal et al., 2007). These reductions showed no sex‐specific effects. However, despite both sexes having an overall survival exceeding 60% in both treatments, suggesting a harmful but non‐lethal effect of heat stress (Green et al., 2019), survivors from the high‐temperature treatment displayed severe sex‐specific effects on reproduction. In females, heat stress only reduced mating probability, while male exposure to the elevated temperature treatment caused multiple adverse effects: prolonged copulations which may increase predation risk (Kaitala & Wiklund, 1994); a 39.2% drop in their mates' egg production; and a catastrophic 97.7% reduction in fertility, indicating that this temperature exceeds the thermal fertility limit in this species (TFL_80_; the temperature at which 80% of the population become sterile; Parratt et al., 2021; Bretman et al., 2024). Male sterility was linked to disrupted fertilising eupyrene sperm production or transfer, which did not recover after remating. These negative effects of heat stress on male fertility and behaviour, in conjunction with a reduction in female mating success, could negatively impact population growth. Our findings reinforce the need to assess sex‐specific reproductive thermal sensitivity at temperatures thought to optimise somatic traits, as ignoring these effects may lead to overly optimistic climate change forecasts (Parratt et al., 2021).

Developmental heat stress profoundly affected male fertility, resulting in the absence of eupyrene sperm in the spermatophore. Males failed to recover fertility in a subsequent mating, presumably as eupyrene sperm production or transfer was still disrupted. Non‐fertilising apyrene sperm number and length were also reduced, although the fitness impact of this is unclear. The mechanism impairing spermatogenesis is unknown, although temperature stress causes disruption to spermatid elongation, sperm transfer and increased sperm cell death in *Drosophila* (David et al., 2005; Gandara & Drummond‐Barbosa, 2023; Green et al., 2019). Our results quantitatively show differential thermal sensitivity between sperm types (Iossa et al., 2019), with eupyrene spermatogenesis, which begins earlier in development, being more susceptible to heat stress than apyrene spermatogenesis (Friedländer, 1997). DNA damage, which is frequently associated with heat‐induced sterility (Kurhanewicz et al., 2020), would only affect eupyrene sperm, so may also contribute to this differential thermal sensitivity. Similar effects in two moth species, with *Plodia interpunctella* having shorter sperm lengths of both types (Iossa et al., 2019) and *Lobesia botrana* having fewer eupyrene sperm bundles (Iltis et al., 2020), suggest a broad pattern of disrupted fertilising sperm production under warming climates.

Behavioural mating traits also displayed sex‐specific sensitivity to heat stress. Copulation duration in *P. napi* is governed by the male (Wickman, 1985), related to the packaging and transfer of the spermatophore (Bissoondath & Wiklund, 1996). Previous work showed that non‐virgin males take longer to transfer a spermatophore of adequate size, prolonging copulation duration (Kaitala & Wiklund, 1994), particularly in males with reduced body mass (Wiklund & Kaitala, 1995). We expand on this result, showing that exposure to heat stress also extends copulation duration. Temperature‐induced disruptions to spermatophore production may also partially explain why males reared at 29°C reduced their mates' fecundity, as spermatophores provide valuable nutrients (e.g. nitrogen) obtained from larval feeding and supplemented during adulthood (Karlsson, 1998; Lederhouse et al., 1990), affecting female egg production (Karlsson, 1998; Larsdotter Mellström & Wiklund, 2015; Wedell & Karlsson, 2003; Wiklund & Kaitala, 1995). As we did not measure spermatophore nutrient content or size, the exact mechanism underlying reduced fecundity and increased copulation durations remains unclear. Regardless, females can experience fitness costs from extended copulations (e.g. sexual conflict, Andersson et al., 2004; predation and disease transmission, Partridge, 1987; Smith, 2012), which may select for better female discrimination of males to avoid the costs associated with mating with sterile males. However, as our choice experiments showed no significant difference in male mating success between treatments, and research on the speckled wood butterfly, *Pararge aegeria* (L.), found no evidence that females can discriminate between virgin and recently mated males (Vande Velde et al., 2011), evidence for female discrimination between males is limited. Additionally, while females that first mate with a sterile male may be able to increase fecundity by remating with a non‐stressed male, non‐stressed males had a near‐significant decrease in fertility in the second mating, corroborating previous work in *P. napi* (Bissoondath & Wiklund, 1996) and other Lepidoptera species (Svärd & Wiklund, 1986). This ejaculate limitation likely restricts female potential to increase fecundity through remating, which could further impact population growth (Wedell et al., 2002).

In contrast to male mating success, elevated temperatures reduced female mating success under choice conditions. We offer three non‐mutually exclusive hypotheses to explain this effect. First, females exposed to stressful developmental temperatures may be physiologically compromised, reducing their willingness to copulate to conserve energy. Second, heat stress may disrupt females' ability to detect or process male sex pheromones released during courtship (e.g. citral; Andersson et al., 2004, 2007), required for mating acceptance in this species. Third, mate choice in this species is dictated by females (Andersson et al., 2007), but courtship initiation is male‐driven (Wedell, 2010). Males may be less willing to court and mate with heat‐stressed females, who are smaller and slower to remate (Wedell & Cook, 1999), and heat‐stressed females may display fewer behaviours that signal mating receptivity. Irrespective of the mechanism, decreases in the mating pool can reduce effective population size, increasing the potential for population extinction as the climate warms (Frankham, 1995). While mating behaviour may be affected by flight constraints in the cages used, a quantitative synthesis of studies examining the effect of flight cage on mating behaviour in the butterfly *B. anynana* found that mating outcomes were only affected by flight cage when sex ratios were biased (Nieberding & Holveck, 2017). Our experimental design had an equal sex ratio in all cages, so, assuming a similar response to cage size in *P. napi*, it is unlikely that cage size differentially affected our results.

Limitations of our interpretations include applying constant heat stress throughout larval development until adult eclosion and examining one population. First, constant stressful temperatures may overestimate the thermal sensitivity of reproduction if thermal fluctuations over shorter time periods allow stress recovery (Ma et al., 2021). However, a recent meta‐analysis found that treatments that periodically fluctuate around the constant temperature value can be as detrimental to phenotypic outcomes as high constant thermal stress, and that constant developmental temperatures can accurately predict performance under natural temperature conditions (Raynal et al., 2022). Moreover, our experimental conditions may become more prevalent as both mean temperature and heatwave duration, frequency and intensity are predicted to increase (IPCC, 2014). Second, our work examined a single population of one species. For many non‐reproductive traits, local adaptation to regional thermal conditions can occur, including in *P. napi* (Günter et al., 2020). While our results suggest substantial potential for reduced population size in this population, future work should examine other temperature scenarios and other *P. napi* populations to better understand the role of heat stress on reproductive traits and consequences for population persistence. Additionally, given that the majority of European Lepidopteran species are predicted to be negatively affected by increasing temperatures due to climate change (Settele et al., 2008), replicating this study in other species would also be valuable.

## 5 CONCLUSIONS

We show that sublethal temperatures that generate the fastest development rate have detrimental effects on reproduction in *P. napi*. This underscores the importance of examining multiple traits that influence population growth rate when predicting population dynamics in response to climate change. Additionally, each reproductive trait displayed sex‐specific thermal sensitivities. Heat‐induced male sterility due to the disruption of eupyrene sperm production or transfer is likely to be compounded by the negative effects of temperature stress on female mating success and the extended copulation duration of heat‐stressed males. Overall, the repercussions of reproductive thermal stress on population persistence will depend on the intimate interactions between the sexes, such that investigating the thermal sensitivity of multiple aspects of reproduction, across populations and species, is necessary for an improved understanding of species vulnerability to climate change.

## AUTHOR CONTRIBUTIONS

R. R. Snook conceived the ideas; R. R. Snook, K. Gotthard and C. Wiklund designed the methodology; D. Trojmar, C. Wiklund, R. R. Snook and M. Q. R. Pembury Smith collected the data; M. Q. R. analysed the data; M. Q. R. and R. R. Snook led the writing of the manuscript. All authors contributed critically to the drafts and gave final approval for publication.

## CONFLICT OF INTEREST STATEMENT

The authors declare no conflicts of interest.

## STATEMENT ON INCLUSION

Our study brings together a number of authors in the country where the study was carried out. All authors were engaged early on with the research to ensure that all perspectives were considered from the onset. Whenever relevant, literature published by scientists from the local region was cited.

## Supporting information


**Table S1:** Description of the statistical models used for the analysis.
**Table S2:** Model selection for examining pupal survival using log‐likelihood.
**Table S3:** Model selection for examining adult body weight using log‐likelihood.
**Table S4:** Model selection for examining mating success using log‐likelihood.
**Table S5:** Model selection for examining egg production and fertility in 2021 using log‐likelihood.
**Table S6:** Model selection for examining egg production and fertility on day 3 between 2021 and 2022 using log‐likelihood.
**Table S7:** ANOVA examining copulation duration following an additional copulation with a benign female.
**Table S8:** Summary data of all traits measured in males and females exposed to two different thermal conditions during development.

## Data Availability

Data available from the Dryad Digital Repository: https://doi.org/10.5061/dryad.dfn2z35cj (Pembury Smith et al., 2025).
