# Supplementary material for: Sex‐specific variation in thermal sensitivity has multiple negative effects on reproductive trait performance
Source: J Anim Ecol. 2025 Mar 18;94(5):943–57. doi: 10.1111/1365-2656.70026 (PMC12056345; doi:10.1111/1365-2656.70026)
Supplement: Supplementary file 1 — Table S1: Description of the statistical models used for the analysis. Table S2: Model selection for examining pupal survival using log‐likelihood. Table S3: Model selection for examining adult body weight using log‐likelihood. Table S4: Model selection for examining mating success using log‐likelihood. Table S5: Model selection for examining egg production and fertility in 2021 using log‐likelihood. Table S6: Model selection for examining egg production and fertility on day 3 between 2021 and 2022 using log‐likelihood. Table S7: ANOVA examining copulation duration following an additional copulation with a benign female. Table S8: Summary data of all traits measured in males and females exposed to two different thermal conditions during development. [file JANE-94-943-s001.docx]

**Supplementary Material**

**Supplementary Table S1. Description of the statistical models used for the analysis.** “Female Temp” is the female rearing temperature (22°C or 29°C), “Male Temp” is the male rearing temperature (22°C or 29°C), “Temperature Treatment” describes the temperature treatment (22°C or 29°C), “Year” describes the experimental year (2021 or 2022), “Day” describes the experimental day (day 3 or day 6), “Eggs” is the number of eggs produced, “Mating Episode” describes whether it was an initial mating or a remating. “Male identity” is the individual male. “Family” is the distribution of the response variable in the model. “*X^2^*”, “*F*” and “*p*” are the model statistics for the covariate included in the model.

| Variable of interest | Model | Family | Random effect variance ± SE | Covariate | *X^2^/F* | *p* |
| --- | --- | --- | --- | --- | --- | --- |
| Eclosion | Temperature Treatment*Sex + Year | Binomial |  |  |  |  |
| Body Weight | Temperature Treatment*Sex + Year | Gaussian |  |  |  |  |
| Mating Success | Female Temp*Male Temp + Year | Generalised poisson |  |  |  |  |
|  |  |  |  |  |  |  |
| Copulation Duration Initial Mating | Female Temp*Male Temp + Male Weight + Female Weight + (1\|Flight Cage) | Gaussian | 12.800 ± 3.583 | Female Weight | 0.079 | 0.779 |
|  |  |  |  | Male Weight | 1.791 | 0.181 |
| Egg Number (Day 3 vs Day 6) | Female Temp*Male Temp + Day + Male Weight + Female Weight | Gaussian |  | Female Weight | 1.233 | 0.269 |
|  |  |  |  | Male Weight | 0.205 | 0.652 |
| Larval Number (Day 3 vs Day 6) | Female Temp*Male Temp + Day + Male Weight + Female Weight + Eggs | Gaussian |  | Female Weight | 0.893 | 0.345 |
|  |  |  |  | Male Weight | 4.871 | 0.028 |
|  |  |  |  | Eggs | 59.797 | <0.001 |
| Egg Number Day 3 (2021 vs 2022) | Female Temp*Male Temp + Year + Male Weight + Female Weight | Gaussian |  | Female Weight | 1.775 | 0.185 |
|  |  |  |  | Male Weight | 0.301 | 0.584 |
| Larval Number Day 3 (2021 vs 2022) | Female Temp*Male Temp + Year + Male Weight + Female Weight + Eggs | Gaussian |  | Female Weight | 0.234 | 0.629 |
|  |  |  |  | Male Weight | 0.102 | 0.750 |
|  |  |  |  | Eggs | 140.754 | <0.001 |
| Copulation Duration Initial vs Remating | Female Temp*Male Temp*Mating Episode + Male Weight + Female Weight + (1\| Male Identity) | Gaussian | 228.700 ± 15.120 | Female Weight | 1.094 | 0.296 |
|  |  |  |  | Male Weight | 2.393 | 0.122 |
| Egg Number Initial vs Remating | Female Temp*Male Temp*Mating Episode + Male Weight + Female Weight + (1\| Male Identity) | Gaussian | <0.001 ± 0.001 | Female Weight | 0.508 | 0.476 |
|  |  |  |  | Male Weight | 0.081 | 0.776 |
| Larval Number Initial vs Remating | Female Temp*Male Temp*Mating Episode + Male Weight + Female Weight + Eggs + (1\| Male Identity) | Gaussian | <0.001 ± 0.001 | Female Weight | 0.012 | 0.914 |
|  |  |  |  | Male Weight | 0.533 | 0.465 |
|  |  |  |  | Eggs | 0.370 | 0.543 |
| Eupyrene Number | Female Temp + Male Temp + Male Weight + Female Weight | Gaussian |  | Female Weight | 0.109 | 0.743 |
|  |  |  |  | Male Weight | 1.498 | 0.233 |
| Apyrene Number | Female Temp + Male Temp + Male Weight + Female Weight | Gaussian |  | Female Weight | 0.864 | 0.361 |
|  |  |  |  | Male Weight | 0.205 | 0.654 |
| Apyrene Length | Female Temp + Male Temp + Male Weight + Female Weight + (1\| Male Identity) | Gaussian | 0.001 ± 0.036 | Female Weight | 0.048 | 0.827 |
|  |  |  |  | Male Weight | 10.865 | <0.001 |

**Supplementary Table S2: Model selection for examining pupal survival using log likelihood.** A full model was compared to a simplified model using the *anova()* function. Akaike Information Criterion *“*AIC” measures the relative quality of a statistical model given the data set used by balancing goodness of fit and model complexity. Log-likelihood “LogLik” measures how likely it is that the model would have produced the observed data given model parameters. “2*Deviance” is the test statistic which is compared to a critical value from a chi-squared distribution to determine whether the difference in fit is statistically significant. Degrees of freedom “*df*” refers to difference in the number of parameters between models. The “*p”* value indicates whether the difference in the model fit is statistically significant. Model comparison was conducted on generalised linear models with binomial error distributions representing the effects of temperature, sex and year on pupal survival. The response variable was entered as a paired ‘cbind(eclosed, not eclosed)’. The final model used is in bold.

| Model | AIC | LogLik | 2*Deviance | *df* | *p* |
| --- | --- | --- | --- | --- | --- |
| **Temperature*Sex + Year** | **53.565** | **-21.782** |  |  |  |
| Temperature*Sex*Year | 57.708 | -20.854 | 1.857 | 3 | 0.603 |

**Supplementary Table S3: Model selection for examining adult body weight using log likelihood.** A full model was compared to a simplified model using the *anova()* function. Akaike Information Criterion *“*AIC” measures the relative quality of a statistical model given the data set used by balancing goodness of fit and model complexity. Log-likelihood “LogLik” measures how likely it is that the model would have produced the observed data given model parameters. Sum of Squares “SumSq” describes the deviance in the amount of variance explained by each model and is used to identify whether adding additional parameters improves the fit of the model. The “*F”* value is the test statistic for the likelihood ratio test which is used to compare the two models. Degrees of freedom “*df*” refers to the difference in the number of parameters between models. The “*p”* value indicates whether the difference in the model fit is statistically significant. Analysis was conducted on linear model representing the effects of temperature, sex and year on adult body weight. The final model is in bold.

| Model | AIC | LogLik | SumSq | *F* | *df* | *p* |
| --- | --- | --- | --- | --- | --- | --- |
| **Temperature*Sex + Year** | **-2273.472** | **1142.736** |  |  |  |  |
| Temperature*Sex*year | -2270.678 | 1144.339 | <0.001 | 1.049 | 3 | 0.371 |

**Supplementary Table S4: Model selection for examining mating success using AIC and LogLik.** A full model was compared to a simplified model using the *anova()* function. Akaike Information Criterion *“*AIC” measures the relative quality of a statistical model given the data set used by balancing goodness of fit and model complexity. Log-likelihood “LogLik” measures how likely it is that the model would have produced the observed data given model parameters. “2*Deviance” is the test statistic which is compared to a critical value from a chi-squared distribution to determine whether the difference in fit is statistically significant. Degrees of freedom “*df*” refers to difference in the number of parameters between models. The “*p”* value indicates whether the difference in the model fit is statistically significant. Analysis was conducted on a generalised poisson model representing the effects of male temperature, female temperature and mating episode on mating success. The final model is in bold.

| Model | AIC | LogLik | 2*Deviance | *df* | *p* |
| --- | --- | --- | --- | --- | --- |
| **Male Temperature*Female Temperature + Mating Episode** | **111.453** | **-49.727** |  |  |  |
| Male Temperature*Female Temperature*Mating Episode | 116.449 | -49.224 | 1.005 | 3 | 0.800 |

**Supplementary Table S5: Model selection for examining egg production and fertility in 2021 using log likelihood.** A full model was compared to a simplified model using the *anova()* function. Akaike Information Criterion *“*AIC” measures the relative quality of a statistical model given the data set used by balancing goodness of fit and model complexity. Log-likelihood “LogLik” measures how likely it is that the model would have produced the observed data given model parameters. Sum of Squares “SumSq” describes the deviance in the amount of variance explained by each model and is used to identify whether adding additional parameters improves the fit of the model. The “*F”* value is the test statistic for the likelihood ratio test which is used to compare the two models. Degrees of freedom “*df*” refers to the difference in the number of parameters between models. The “*p”* value indicates whether the difference in the model fit is statistically significant. Analysis was conducted on linear models, representing the effects of male temperature, female temperature and measurement day on (i) egg number and (ii) larval number (fertility) post initial copulation. The covariates “Male Weight” and “Female Weight” are included in all models and the covariate “Eggs” (the number of eggs produced) is included in models evaluating larval number. The final model is in bold.

1. Egg number

| Model | AIC | LogLik | SumSq | *F* | *df* | *p* |
| --- | --- | --- | --- | --- | --- | --- |
| **Male temperature*Female Temperature + Year + Male Weight + Female Weight** | **757.860** | **-370.930** |  |  |  |  |
| Male Temperature*Female Temperature *Year + Male Weight + Female Weight | 757.835 | -368.917 | 32.688 | 1.269 | 3 | 0.287 |

1. Larval number (fertility)

| Model | AIC | LogLik | SumSq | *F* | *df* | *p* |
| --- | --- | --- | --- | --- | --- | --- |
| **Male Temperature*Female Temperature + Day + Male Weight + Female Weight + Eggs** | **1190.000** | **-586.000** |  |  |  |  |
| Male Temperature*Female Temperature*Day + Male Weight + Female Weight + Eggs | 1193.120 | -584.558 | 8.357 | 0.931 | 3 | 0.426 |

**Supplementary Table S6: Model selection for examining egg production and fertility on day 3 between 2021 and 2022 using log likelihood.** A full model was compared to a simplified model using the *anova()* function. Akaike Information Criterion *“*AIC” measures the relative quality of a statistical model given the data set used by balancing goodness of fit and model complexity. Log-likelihood “LogLik” measures how likely it is that the model would have produced the observed data given model parameters. Sum of Squares “SumSq” describes the deviance in the amount of variance explained by each model and is used to identify whether adding additional parameters improves the fit of the model. The “*F”* value is the test statistic for the likelihood ratio test which is used to compare the two models. Degrees of freedom “*df*” refers to the difference in the number of parameters between models. The “*p”* value indicates whether the difference in the model fit is statistically significant. Analysis was conducted on linear models, representing the effects of male temperature, female temperature and experimental year on (i) egg number and (ii) larval number (fertility) from days 1 – 3 post initial copulation. The covariates “Male Weight” and “Female Weight” are included in all models and the covariate “Eggs” (the number of eggs produced) is included in models evaluating larval number. The final model is in bold.

1. Egg number

| Model | AIC | LogLik | SumSq | *F* | *df* | *p* |
| --- | --- | --- | --- | --- | --- | --- |
| **Male Temperature*Female Temperature + Year + Male Weight + Female Weight** | **636.810** | **-310.405** |  |  |  |  |
| Male Temperature*Female Temperature*Year + Male Weight + Female Weight | 640.365 | -309.183 | 13.624 | 0.762 | 3 | 0.518 |

1. Larval number (fertility)

| Model | AIC | LogLik | SumSq | *F* | *df* | *p* |
| --- | --- | --- | --- | --- | --- | --- |
| **Male Temperature*Female Temperature + Year + Male Weight + Female Weight** | **1067.151** | **-524.575** |  |  |  |  |
| Male Temperature*Female Temperature*Year + Male Weight + Female Weight + Eggs | 1006.007 | -521.004 | 966.300 | 2.246 | 3 | 0.086 |

**Supplementary Table S7: ANOVA examining copulation duration following an additional copulation with a benign female.** Male weight and the weight of the male’s second mate were included as covariates, and male identity was included as a random effect to account for repeated measures (Table S1). Significant effects are in bold.

| Variable | *X^2^* | *df* | *p* |
| --- | --- | --- | --- |
| Male Treatment | 1.800 | 1 | 0.180 |
| Female Treatment | 0.245 | 1 | 0.621 |
| **Mating Episode** | **5.091** | **1** | **0.024** |
| Male Treatment*Female Treatment | 0.004 | 1 | 0.950 |
| Male Treatment*Mating Episode | 0.251 | 1 | 0.617 |
| Female Treatment*Mating Episode | 1.108 | 1 | 0.293 |
| Male Treatment*Female Treatment*Mating Episode | 1.337 | 1 | 0.248 |

**Supplementary Table S8:** **Summary data of all traits measured in males and females exposed to two different thermal conditions during development.** Butterflies were exposed to one of two constant developmental thermal conditions (22°C or 29°C). The experiment was conducted twice over two consecutive years: 2021 and 2022. The effect of temperature on five different traits was examined during both years: pupal survival, body weight, mating success (the frequency of each temperature treatment from a mating pair measured from the first 50% of matings), egg production (egg number; measured 3 days after mating) and larval number (fertility). In 2021 we also examined the effect of measurement day (3 and 6) on both egg number and fertility. In 2022 we also examined the effect of temperature on a number of additional traits: copulation duration (during an initial mating and during an additional mating with a 22°C-reared female 2 days after the initial mating), recovery (egg and larval number during an initial mating and following an additional mating with a 22°C-reared female 2 days after the initial mating), eupyrene sperm number, apyrene sperm number and apyrene sperm length. No measures of eupyrene sperm length were taken as males from the 29°C treatment did not produce any eupyrene sperm. For the above table, column title describes the temperature treatment mating combination. The first number represents the temperature treatment of the female and the second number represents the temperature treatment of the male (e.g. 22F22M describes 22°C-reared females paired with 22°C-reared males). For the below table, column title describes the temperature treatment the individual was exposed to during development.

|  |  |  | 22F22M |  |  | 22F29M |  |  | 29F22M |  |  | 29F29M |  |
| --- | --- | --- | --- | --- | --- | --- | --- | --- | --- | --- | --- | --- | --- |
|  |  | Mean | Error | n | Mean | Error | n | Mean | Error | n | Mean | Error | n |
| Mating success |  |  | 6.86 | 47 |  | 6.16 | 38 |  | 5.29 | 28 |  | 3.61 | 13 |
| Copulation duration initial mating (mins) |  | 99.21 | 5.23 | 24 | 115.85 | 5.66 | 26 | 97.25 | 7.40 | 24 | 116.14 | 6.87 | 7 |
| Effect of day on egg number | Day 3 | 51.16 | 7.06 | 25 | 26.82 | 3.71 | 22 | 42.88 | 5.68 | 17 | 46.00 | 6.74 | 11 |
|  | Day 6 | 88.72 | 12.83 | 25 | 41.45 | 9.07 | 22 | 82.00 | 12.41 | 17 | 46.45 | 11.78 | 11 |
| Effect of day on larval number | Day 3 | 28.28 | 4.34 | 25 | 1.05 | 0.34 | 22 | 21.65 | 3.67 | 17 | 0.91 | 0.81 | 11 |
|  | Day 6 | 39.24 | 6.00 | 25 | 2.45 | 1.57 | 22 | 31.12 | 4.28 | 17 | 1.55 | 0.49 | 11 |
| Effect of year on day 3 egg number | 2021 | 46.27 | 6.80 | 33 | 27.12 | 3.58 | 25 | 45.00 | 6.89 | 19 | 41.62 | 6.69 | 13 |
|  | 2022 | 47.31 | 9.81 | 13 | 20.92 | 2.74 | 13 | 36.19 | 10.91 | 11 | 23.00 | 4.59 | 9 |
| Effect of year on day 3 larval number | 2021 | 24.18 | 3.71 | 33 | 1.08 | 0.32 | 25 | 26.11 | 5.65 | 18 | 0.77 | 0.69 | 13 |
|  | 2022 | 31.92 | 6.45 | 13 | 0.00 | 0.00 | 13 | 22.00 | 8.04 | 11 | 6.00 | 3.97 | 9 |
| Copulation duration remating (mins) | Initial | 80.86 | 2.48 | 7 | 111.64 | 6.04 | 11 | 99.22 | 13.89 | 9 | 111.33 | 3.84 | 3 |
|  | Remate | 121.71 | 7.95 | 7 | 164.09 | 16.85 | 11 | 114.67 | 15.60 | 9 | 184.00 | 28.69 | 3 |
| Egg number remating | Initial | 44.67 | 15.01 | 3 | 21.20 | 6.09 | 5 | 32.25 | 4.78 | 4 | 13.67 | 8.01 | 3 |
|  | Remate | 86.33 | 32.87 | 3 | 7.60 | 1.96 | 5 | 37.25 | 17.61 | 4 | 25.00 | 16.50 | 3 |
| Larval number remating | Initial | 32.00 | 10.15 | 3 | 0.00 | 0.00 | 5 | 23.00 | 4.36 | 4 | 0.00 | 0.00 | 3 |
|  | Remate | 18.67 | 9.56 | 3 | 0.00 | 0.00 | 5 | 4.50 | 4.17 | 4 | 0.00 | 0.00 | 3 |
| Eupyrene number |  | 8529 | 847 | 10 | 0 | 0 | 11 | 8988 | 975 | 9 | NA | NA | NA |
| Apyrene number |  | 56294 | 7489 | 10 | 2579 | 862 | 11 | 68256 | 7335 | 9 | NA | NA | NA |
| Apyrene length (mm) (individual) |  | 0.45 | 0.01 | 66(11) | 0.22 | 0.02 | 43(11) | 0.45 | 0.01 | 54(9) | NA | NA | NA |

|  |  | Male Temperature | | | | | | Female Temperature | | | | | |
| --- | --- | --- | --- | --- | --- | --- | --- | --- | --- | --- | --- | --- | --- |
|  |  | 22°C | | | 29°C | | | 22°C | | | 29°C | | |
|  |  | Mean | Error | *n* | Mean | Error | *n* | Mean | Error | *n* | Mean | Error | *n* |
| Pupal survival | Yes |  |  | 251 |  |  | 160 |  |  | 247 |  |  | 164 |
|  | No |  |  | 87 |  |  | 111 |  |  | 69 |  |  | 83 |
| Body weight (mg) |  | 0.07 | 0.001 | 103 | 0.07 | 0.001 | 72 | 0.07 | 0.001 | 110 | 0.07 | 0.001 | 65 |
